# All Alone We Go Faster, Together We Go Further: The Necessary Evolution of Professional and Elite Sporting Environment to Bridge the Gap Between Research and Practice

**DOI:** 10.3389/fspor.2020.631147

**Published:** 2021-01-27

**Authors:** Franck Brocherie, Adam Beard

**Affiliations:** ^1^Laboratory Sport, Expertise and Performance (EA 7370), French Institute of Sport (INSEP), Paris, France; ^2^High Performance Unit, Chicago Cubs Major League Baseball, Chicago, IL, United States

**Keywords:** athletes, biomedical–standards, evidence-based/evidence-informed practice, organization & administration, decision making, humans

## Setting the Stage

**Figure 1:**
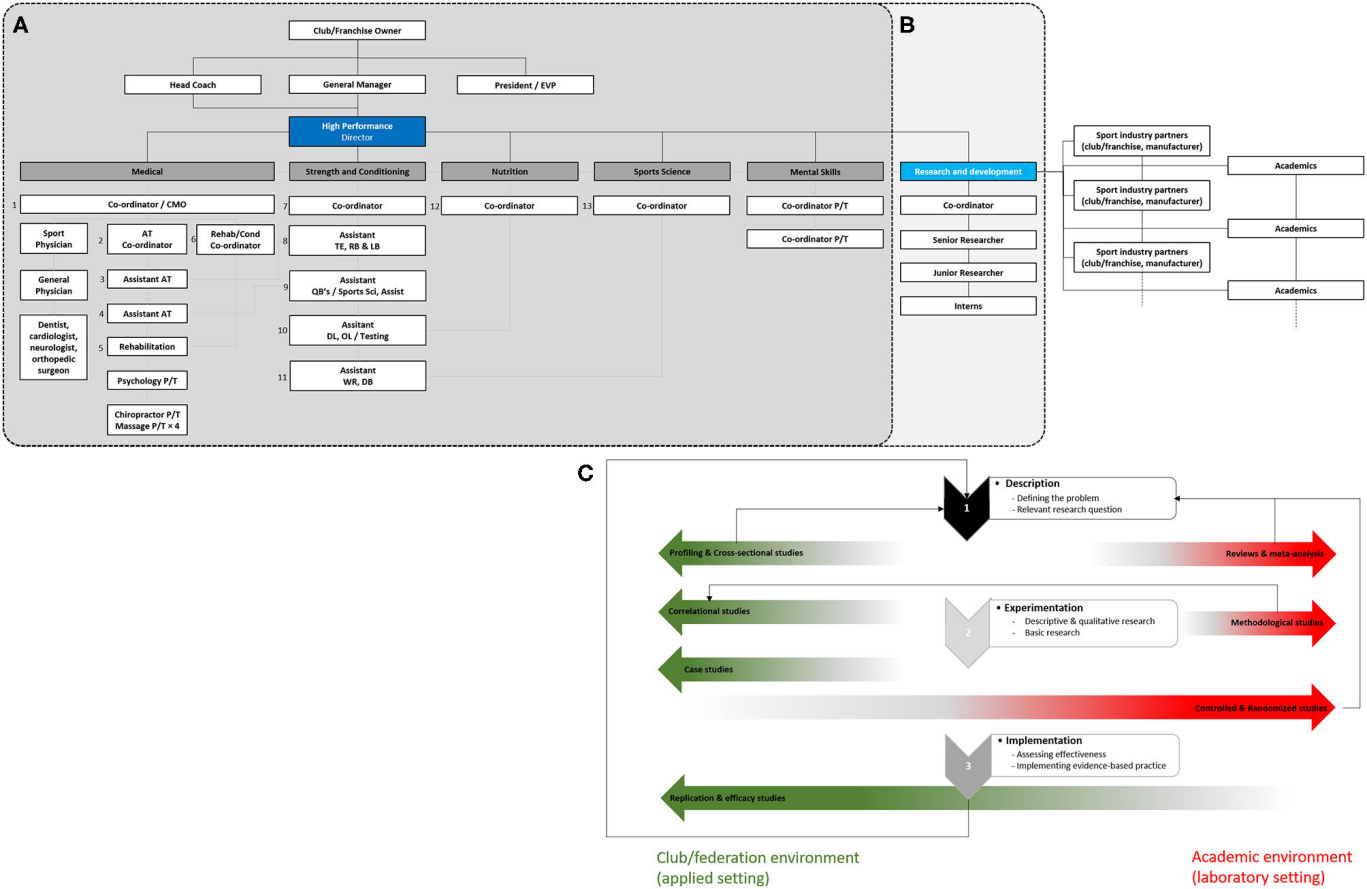
The actual **(A)** and proposed **(B)** performance support model and its applied research process **(C)**. AT, athletic trainer; CMO, chief medical officer; DB, defensive back; DL, defensive line; EVP, executive vice president; LB, linebacker; OL, offensive line; P/T, physical therapist; QB, quarterback; RB, running back; TE, tight end; WR, wide receiver.

The landscape of the professional and elite sport has changed enormously in recent years, with clubs/franchises and national federations performance support operating through specialized background staff roles. Although not uniformly embraced across all sports and countries, the expansion of such a model has led to the emergence of a managing position—generally termed performance director (Buchheit and Carolan, 2019)—to organize and supervise all the sports science and sports medicine servicing areas accessible to the head coach (and/or his technical staff) and athletes. The scientific support staffing base includes full-time sport scientists, physiologists, biomechanists, nutritionists, psychologists, and even more recently statisticians/data scientists, with some additional part-time input from expert/academic consultants (e.g., neuroscientists). Depending of the size and culture of the clubs/federations, a medical department covers the medical care and therapy related to training and competition, as well as the involvement of professional specialists for health management (Dijkstra et al., 2014). As an example, a National Football League (NFL) staff generally comprised five departments and as large as 13 full-time employees under the umbrella of the performance director (Figure 1A). All these departments operate in synergy and also “independently” with appropriate autonomy at times, with the performance director orchestrating the “front lines” in a holistic and comprehensive manner toward a common performance goal.

The impetus to drive a performance support model is directly related to assisting the coaching/front office staff on strategies to understand what winning looks like through analysis of key performance indicators and metrics (Halson et al., 2019). The performance model employs analysis technologies (e.g., global positioning system with embedded tri-axial accelerometers, gyroscope and magnetometer, wearable sensors) and scientific advances (e.g., innovative training or nutritional strategies) (Malone et al., 2019) to enhance player performance and maximize player availability (Drew et al., 2017) while maintaining their health integrity through an integrated health management system (Dijkstra et al., 2014). Despite the growing number of clubs/federations employing this approach, there are still many who do not choose to see this model as the vehicle to progress. Although this has been widely addressed (Bishop, 2008; Dijkstra et al., 2014; Buchheit, 2016, 2017; Coutts, 2016, 2017; McCall et al., 2016; Eisenmann, 2017; Nassis, 2017; Halperin, 2018; Sandbakk, 2018, 2019; Fullagar et al., 2019; Halson et al., 2019), here, the present opinion proposes to discuss past, actual, and new issues faced by the practitioners and researchers that are at the front line of professional and elite sport in order to reinforce the necessary evolution of professional squads and federations to stay at the cutting edge of performance optimization.

## Integration of the Performance Model Into Traditional Settings

Modern professional and elite sport has gained an interest in creating athlete-centered structures (e.g., Boston Celtics Auerbach training center, Ultimate Fighting Championship's performance institute in Las Vegas, Aspire Academy in Doha, Chicago Cubs' Arizona spring training performance center, and Wrigley field high-performance facility), which include state-of-the-art sport science facilities and material for performance optimization. Because the margin between winning and losing is tiny (Davison et al., 2009), such environments take into account all the factors surrounding athlete's performance, health, and well-being.

In order to provide effective evidence-based, performance-oriented, and science-driven practices in sports science and sports medicine support, a positive integration is paramount, implying the organizational direction [i.e., owner, chief executive officers (CEOs), head coach, front office] to recognize and believe in the performance model and then favor the interaction between each department. As such, and because this has been reported to be a critical barrier (Fullagar et al., 2019), particular attention must be carried on ensuring that there is alignment between leadership/ownership and the performance team. This is especially ringing true on the “hands-on” staff (such as coaching, performance, and medical) that should view the overall picture of the organization culture and its performance model and develop coexistence and relationship based on different expertise enabling all staff. A clear holistic process with transparent roles and responsibilities facilitates decision-making regarding the somewhat paradoxal performance optimization and long-term health management (particularly relevant in youth elite sport environments) (Dijkstra et al., 2014).

However, problems may occur if groups within the club/federation are not open to new innovative ideas and scientific methodologies based on evidence-based practices to the optimization of player performance and health. Fixed mindsets not only create problems for the integration of the performance model (Nassis, 2017) but also may create silos between the performance departments and coaches/front office staff (Eisenmann, 2017; Drust, 2019). Clear goals and expectations with regard to where current practices are at the club/federation will help to plan the evolution of the “*here and now—winning today”* and the “*how do we maintain and sustain winning—success”*. If early adopter or innovator profiles would be helpful for compliance and acceptance (Nassis, 2017), in all cases, communication and time are keys to convince (unwilling) head coaches and organizational direction. However, because professional and elite sport setting is result-driven, time is lacking to install a confident working environment, where the worst scenario (i.e., losing consecutive matches) inevitably conducts to head coach eviction, thereby affecting the performance process (Drust, 2019).

In order to convince reluctant groups within the club/federation of the benefits from a sports science and sports medicine support model, the recruited performance director must have multiple strings to his/her bow. Based on our own experience, having a mix of practical (playing experience and/or backroom staff) and theoretical knowledge to ensure a clear understanding of the scientific prerequisite is a helpful asset to assist leaders such as the head coach (Bishop, 2008) by using similar language in a mutually respectful manner. In particular, having a scientific background at the postgraduate level (i.e., ideally having a postgraduate MSc or PhD qualification) would allow the identification (including discrediting poor/false research or pseudoscientific approaches) and adoption of effective evidence-based practices (e.g., targeting few identified areas having a meaningful impact on athletes' performance) that would directly and rapidly impact the decision-making process surrounding sport performance (Buchheit, 2016; Coutts, 2017; Nassis, 2017). Furthermore, such effective and easy to use innovative research-informed, practitioner-led interventions are more likely adopted than disruptive ones (Nassis, 2017) and would open doors for more cooperation. Besides agility and adaptability, additional leadership and interpersonal and communication skills would reinforce the communication needs (Eisenmann, 2017) and drive a centralized operating system that promotes the performance model and club/federation culture. As such, the performance director is the “gatekeeper” of the sports science and sports medicine services, ensuring optimal cooperation while avoiding confusion and pitfalls, notably through open paths of communication between staff.

## Refining the Performance Model

The rapid technological development (and its accompanying regulation adjustments) approved by most leading global sporting organizations, in addition to the increasing demand placed on the athletes, may highlight the important role of sports science and sports medicine staff in modern sport success (or failure). Alongside management leadership and acculturation (Jones et al., 2009), improving athletes' compliance for monitoring and evidence-based methodologies provides the opportunity to reinforce the use of specific devices and supporting strategies. For that, the shared decision-making process (i.e., including three key steps: choice, option, and decision) proposed in sports medicine (Dijkstra et al., 2017; Elwyn et al., 2017) may reduce conflict and participate as education mean for effective and succesful support.

The paradox in professional and elite sport setting is the different timelines requested to ensure key decisions (fast-working process) while promoting the best evidence-based practices (slow-working process) (Coutts, 2016, 2017; McCall et al., 2016). In this view, and because the director of performance may represent the cornerstone of the performance model and would have time for translational concept only, embedding a research and development (R&D) department (under the umbrella of performance director, Figure 1B) would be useful to provide scientific expertise in assessing long-term performance solutions and drive new ideas to improve the decision-making process for day-to-day servicing areas (Coutts, 2016, 2017; McCall et al., 2016; Eisenmann, 2017).

In fact, although developing research partnerships and innovation hubs (McCall et al., 2016) remains valid (see section *Reinforcing the Connection*), bringing researchers and their environment within the same organization is probably the most relevant way to bridge the gap between the “field and the lab” via the development of the triad “athlete–coach–researcher” (Sandbakk, 2018, 2019; Fullagar et al., 2019). Such club/franchise- (e.g., FC Barcelona in soccer, Chicago Cubs in baseball) or organization-embedded research (e.g., Australian, English, French, Norwegian institutes of sport) is generally considered to have greater impact on professional practice (Coutts, 2017). Relocating laboratories and researchers close to the field allows to better understand the constraints that may limit evidence-based practice translation (Bishop, 2008) to identify and conduct relevant ecologically valid applied researches (Reade et al., 2008a,b) that align with the “real-world” needs and perspectives (Jones et al., 2019). Improving the servicing resource with an R&D department would open doors for higher sports science and sports medicine research into applied practice (Fullagar et al., 2019) that may benefit higher education within (Bartlett and Drust, 2020) and outside professional and elite sport.

## Reinforcing the Connection

Refining the performance model with the addition of an R&D department also allows to optimize collaboration with academics (McCall et al., 2016) or other infrastructures from the sport industry (e.g., R&D departments issued from the same or another sport/competition, equipment manufacturers). First, because research questions are established and prioritized by the R&D department (Figures 1B,C), thereby avoiding the common belief from many academics (much more than we think; part of those who believe that having practiced and/or coached at low levels equals head coaches' specific knowledge acquired over years) that head coaches are not sufficiently “brained” to share ideas. One may assume that some brilliant research findings emanated from innovation intuitively developed on the field by some head coaches. As such, adopting integrated knowledge translation models (Boland et al., 2020) involving practitioners in a research agenda would benefit end users through common concepts and vocabulary, the ability to link, exchange, and co-produce knowledge (participatory research, athlete engagement or involvement, and community-based research).

In professional and elite sport settings, proper controlled data collection allows a continuum between servicing and research (Halson et al., 2019) through implementation of intervention to verify a hypothesis (e.g., comparing two training methods). Instead of reinventing the wheel, the research iterative and bidirectional model proposed by Bishop (2008) remain topical. Profiling and cross-sectional studies easily implementable in “real-world” settings would provide values to reviews and meta-analyses to verify the problem identified (Figure 1C). Then, methodological and correlational studies are helpful to set the next steps. In addition, conducting qualitative research such as case study of one or few (elite) athletes (e.g., Brechbuhl et al., 2018; Solli et al., 2020) is one of the pathways bridging the gap between research and practice (Halperin, 2018). This may be an interesting “buy-in” strategy to create a working relationship between practitioners and head coaches (Halperin, 2018), which may result in mutual interests and more demanding research such as laboratory-based experiments (Fullagar et al., 2019). Bearing in mind that poor research (or associated approaches) would discredit all the efforts to support sports science and sports medicine, we believe that even difficult to implement parallel-group (e.g., Beard et al., 2019a,b) or crossover design (e.g., Sandbakk et al., 2015) with appropriate randomization remains possible and provides an opportunity to increase the quality of ecological research in the “real world” (Coutts, 2017; Fullagar et al., 2019). Replication studies must be considered at this stage if basic research has been already conducted. Finally, to truly have an impact on “real-world” settings, effectiveness trials, through replication and efficacy studies in ecological conditions, are imperative to improve quality decision in practice. Despite the reluctance of most journals for a “lack of novelty” (McLoughlin and Drummond, 2017; Nature, 2020), replicating experimental results with or without positive findings would be helpful for researchers and practitioners to decide whether a novel finding is real and large enough to have a practical impact. In this view, the recent coopetition (i.e., simultaneous cooperation and competition) proposal to merge performance data (Ramirez-Lopez et al., 2020) may also provide an alternative to improve sample size and ecological validity of applied research. Some organizations [e.g., FC Barçelona, Sacramento Kings, and Los Angeles Dodgers joint research on modeling players' decision (http://www.sloansportsconference.com/activities/research-papers/2019-research-paper-finalists-posters/) presented at the MIT Sloan Sports Analytics international conference] already take the plunge. Connecting with academics also means to be proactive in research grant application. As such, few initiatives get up. For example, last year, Paris Saint-Germain (PSG) and “Polytechnique” sponsored the “Sport analytics challenge” (https://www.agorize.com/en/challenges/xpsg) that allowed students to submit contributions or projects related to Opta data analysis using Python or R programming language aiming to increase PSG sporting performance. The winner received a 3-year thesis or postdoctoral fellowship (worth €100,000 including tax). Other research opportunities also arose from competition winning bid.

Similarly, major competitions such as the Olympic games often boost scientific support and research initiatives (Skibba et al., 2016), Paris 2024 being the last example with a call for elite sport-related scientific project from the French national agency for research. The flip side of the coin is that it fuels the lust of researchers who are out of sport context, increasing the risk of a setback from the head coaches for the interest of sports science and sports medicine. To avoid this and promote its catalyst effect, the funding stakeholders must carefully control the alignment of research project with its practical application in professional and elite sport setting.

## Conclusion and Summarizing Tips

The necessary evolution of professional and elite sport encompasses embracing better communication based on trust and mutual respect with head coach and management board/team, embedding an R&D department to relocate laboratories and researchers close to the field and reinforce their connection with the “real world” to promote best evidence-based, performance-oriented, and science-driven practices. In order to bridge the gap between research and practice and improve its impact on professional and elite sport setting, key considerations are summarized as follows:

- To improve collaboration with coaches/managers and athletes through
  ▪ Creation of a pleasant work environment,
  ▪ Proper communication (e.g., avoiding silos, eliminating segregation),
  ▪ Rapid information dissemination that is meaningful for the different groups,
  ▪ Staff development (e.g., workshop, newsletter),
  ▪ Favor interaction and critical thinking inside and outside the box.
- To establish trust and building relationships with academics or other sports industry's infrastructures through
  ▪ Integration of laboratory-based materials and researchers within the organization,
  ▪ Development of “win–win” solutions (i.e., interesting and useful) promoting aligned inter- or multi-disciplinary research approach,
  ▪ Improving education material and conferences involving scholars, scientists, practitioners, and/or coaches,
- To improve quality decision in practice through
  ▪ Promotion of the best available evidence at the right time for the right athlete,
  ▪ Implementation of the “integration paradigm” whereby research guides practice, but practice also guides research,
  ▪ Guaranteeing stability, consistency of sports science and medicine support. This may require infrastructure's refinement to maintain effective communication.

## Author Contributions

The authors listed have made substantial, direct and intellectual contribution to the work and approved it for publication.

## Conflict of Interest

The authors declare that the research was conducted in the absence of any commercial or financial relationships that could be construed as a potential conflict of interest.
